# Case Report: Giant Cell Tumor of Tendon Sheath After Breast Augmentation

**DOI:** 10.3389/fonc.2022.878635

**Published:** 2022-06-22

**Authors:** Yu Zhang, Yingying Fan, Hongying Zhang, Hong Bu, Min Chen, Jieliang Yang, Zhang Zhang

**Affiliations:** ^1^ Department of Pathology, West China Hospital, Sichuan University, Chengdu, China; ^2^ Department of Pathology, West China Second University Hospital, Sichuan University/West China Women’s and Children’s Hospital, Chengdu, China; ^3^ Laboratory of Pathology, West China Hospital, Sichuan University, Chengdu, China

**Keywords:** breast, giant cell tumor of tendon sheath, breast augmentation, synovial metaplasia, CSF1

## Abstract

Giant cell tumor of tendon sheath (GCTTS) is a benign tumor. It occurs predominantly in the hands, ankles, and knees. A 39-year-old female presented with GCTTS in the right breast after breast augmentation. There was a clear borderline between the tumor and breast tissue. In terms of morphological appearance, synovial metaplasia could be observed in part of the collagenous capsule. The tumor was moderately cellular and was composed of synovium-like monocytes. The main part of the tumor was blended with nested and scattered xanthomatous cells, lymphocytes, and osteoclast-like giant cells. Hemosiderin granules were distributed in the lesion. Immunohistochemical staining and fluorescence *in situ* hybridization (FISH) analyses were performed. CD68 staining was positive in osteoclast-like giant cells. In addition, neither significant USP6 translocation nor CSF1 translocation was detected by FISH. We hypothesized that the pathogenesis of this rare GCT-TS was based on synovial metaplasia and did not depend on the translocation of classical CSF1.

## Introduction

Giant cell tumor of tendon sheath (GCTTS), also known as tenosynovial giant cell tumor, is a benign tumor. Similar to giant cell tumors of bone (GCTB), GCTTS presents with multinucleated osteoclast-like cells, frequent recurrences, rare metastases, and occurrence of bone erosion in advanced cases (1–3). Differently from GCTB, GCTTS arises from the synovium of joints, bursae, or tendon sheaths (4). Based on the growth pattern and clinical characteristics, GCTTS is classified into localized GCTTS and diffuse GCTTS (5). Although GCTTS is regarded as benign tumors, the recurrence rates of localized GCTTS range from 9 to 17%, and those of diffuse GCTTS range from 33 to 50% (6, 7). Moreover, distant metastasis was reported to occur in a few diffuse GCTTS (6).

Despite differences in the growth pattern between localized GCTTS and diffuse GCTTS, the translocation and the expression of CSF1, which is caused by the fusion of CSF1 and COL6A3, are crucial to tumorigenesis in both subtypes of GCTTS (8, 9). CSF1 secretion attracts macrophages that express CSF1R and may induce the formation of multinucleated giant cells (9).

Currently, surgery is the preferred treatment for GCTTS, and local excision with a negative margin of normal tissue is usually considered adequate therapy (10). In some aggressive cases, extensive resection and radiation therapy are needed (11). Targeted agents can block the CSF1/CSF1R pathway, which is an attractive therapeutic strategy for patients with symptomatic GCTTS (12). Currently, targeted agents that directly block CSF1/CSF1R are in clinical development (12). Moreover, GCTTS patients could benefit from CSF1R inhibitors, even patients with absent CSF1 translocation (13, 14).

In this report, we describe a case of GCTTS in breast with a history of prosthesis implantation and studied the relationship between tumorigenesis and the molecular changes involved in this rare tumor.

## Case Presentation

**Figure 1:**
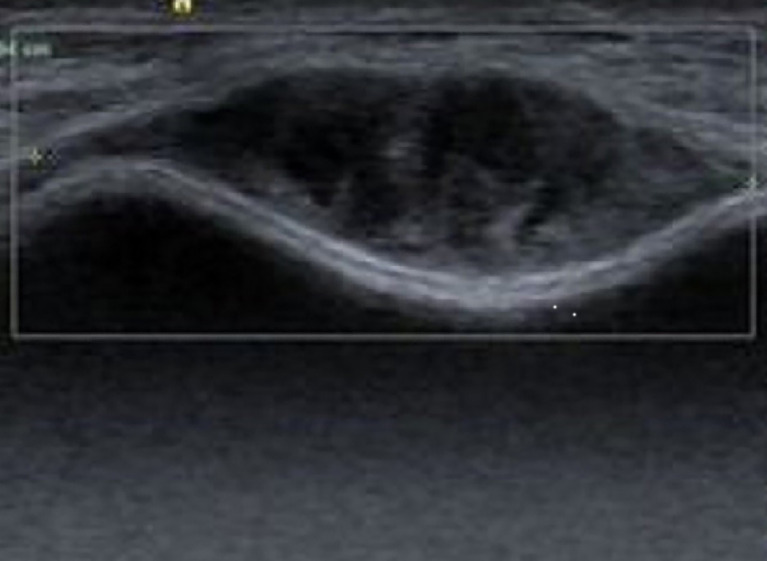
A regular hypoechoic mass on breast ultrasonography.

**Figure 2:**
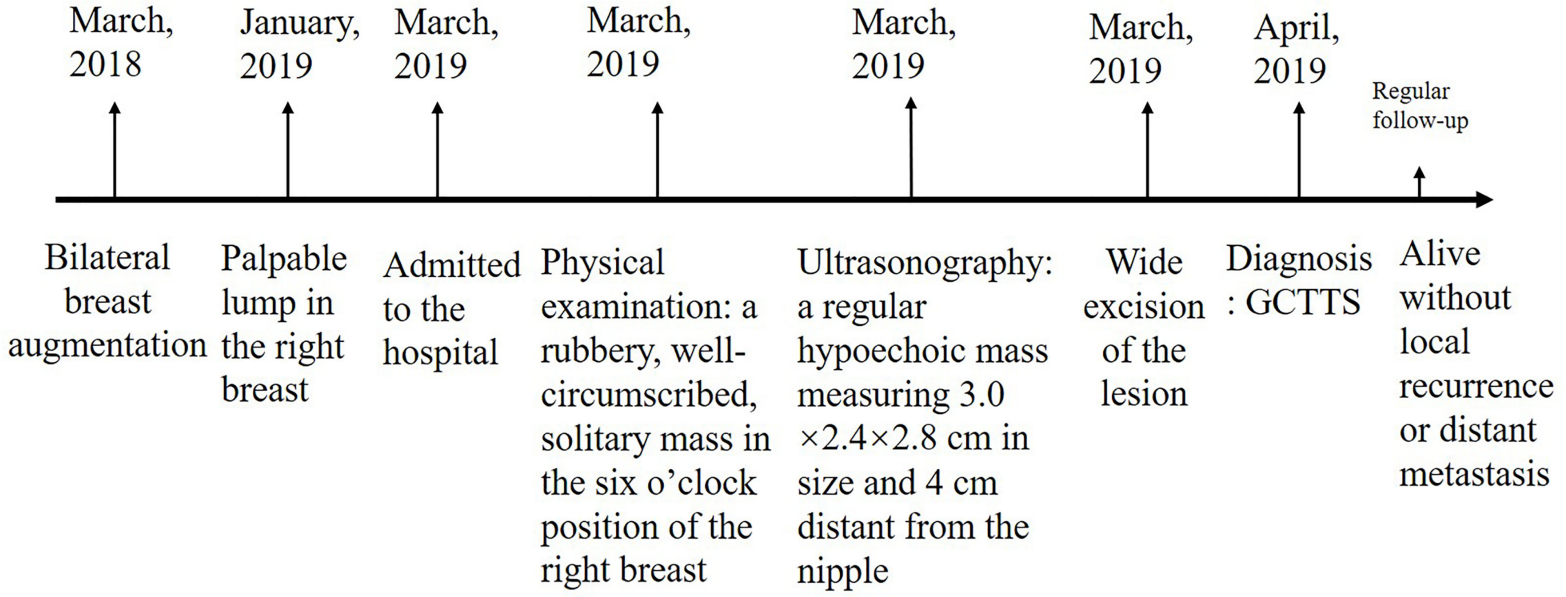
Timeline.

A 39-year-old female was admitted to the hospital with a palpable lump in the right breast for 3 months. A year earlier, the patient underwent bilateral breast augmentation. The physical examination revealed a rubbery, well-circumscribed, solitary mass in the 6 o’clock position of the right breast. An ultrasonography of the breast revealed a regular hypoechoic mass measuring 3.0 cm × 2.4 cm ×2.8 cm in size and 4 cm distant from the nipple (**Figure 1**). There were no palpable axillary nodes. Considering the risk of rupture of prosthesis by biopsy and the well-circumscribed boundary of the tumor, wide excision of the lesion was performed for diagnostic and therapeutic purposes. The timeline is shown in **Figure 2**.

**Figure 3:**
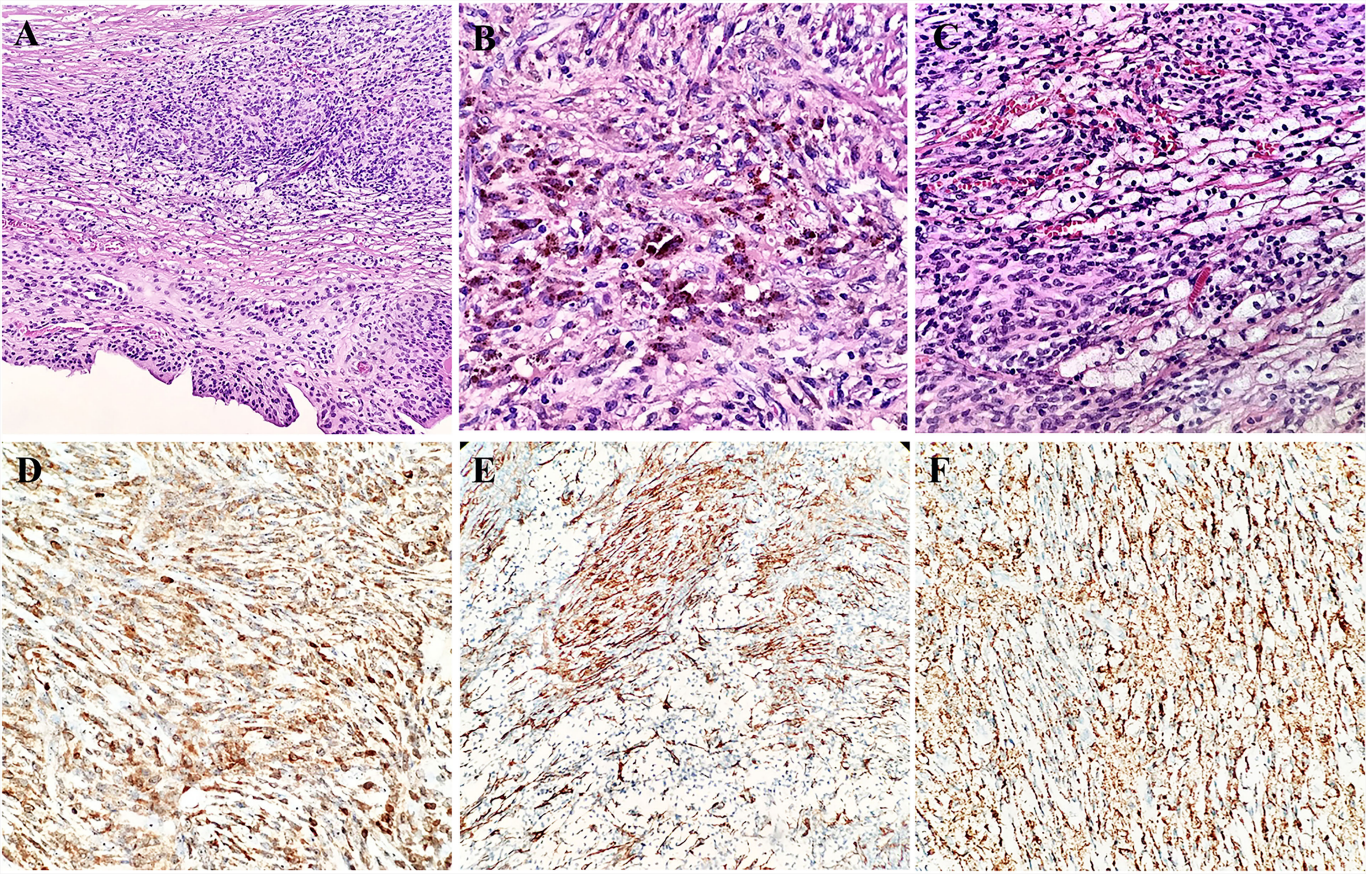
Morphology and immunohistochemistry features of the synovial metaplasia and giant cell tumor of tendon sheath **(A)**. Hemosiderin granule deposition in the lesion **(B)**. Nest-like distribution of xanthomatous cells among monocytes **(C)**. Immunoreactivity for Bcl-2 **(D)**, SMA **(E)**, and CD68 **(F)** in synovium-like cells, mononuclear cells, and osteoclast-like giant cells.

The gross appearance presented as a well-circumscribed mass of 3 cm in diameter. The cut surface was grayish yellow and solitary. Histologically, there was a regular collagenous capsule around the mass. Most areas of the mass had a flat surface covered with finger-like projections or villi. These sheets of cells were composed of many rounded macrophage-like synovial cells and fibroblasts synovial cells and represented as synovial metaplasia (**Figure 3A**). Hemosiderin granules were distributed in the lesion (**Figure 3B**). The tumor was composed of round and polygonal synovium-like monocytes with abundant eosinophilic cytoplasm and rounded and reniform nuclei. The main part of tumor was blended with nested and scattered xanthomatous cells and osteoclast-like giant cells (**Figure 3C**). The osteoclast-like giant cells contained an abundant cytoplasm and many evenly distributed and usually centrally located oval nuclei, some of which contained small nucleoli. Only a few lymphocytes were scattered in the monocytes. Neither seepage of material from prosthesis nor from foreign refractile material was identified in this nodule. Epithelial cells in spindle cell elements were not observed. Cleft-like spaces, rare mitotic figures, and hyalinized stroma could be seen.

**Figure 4:**
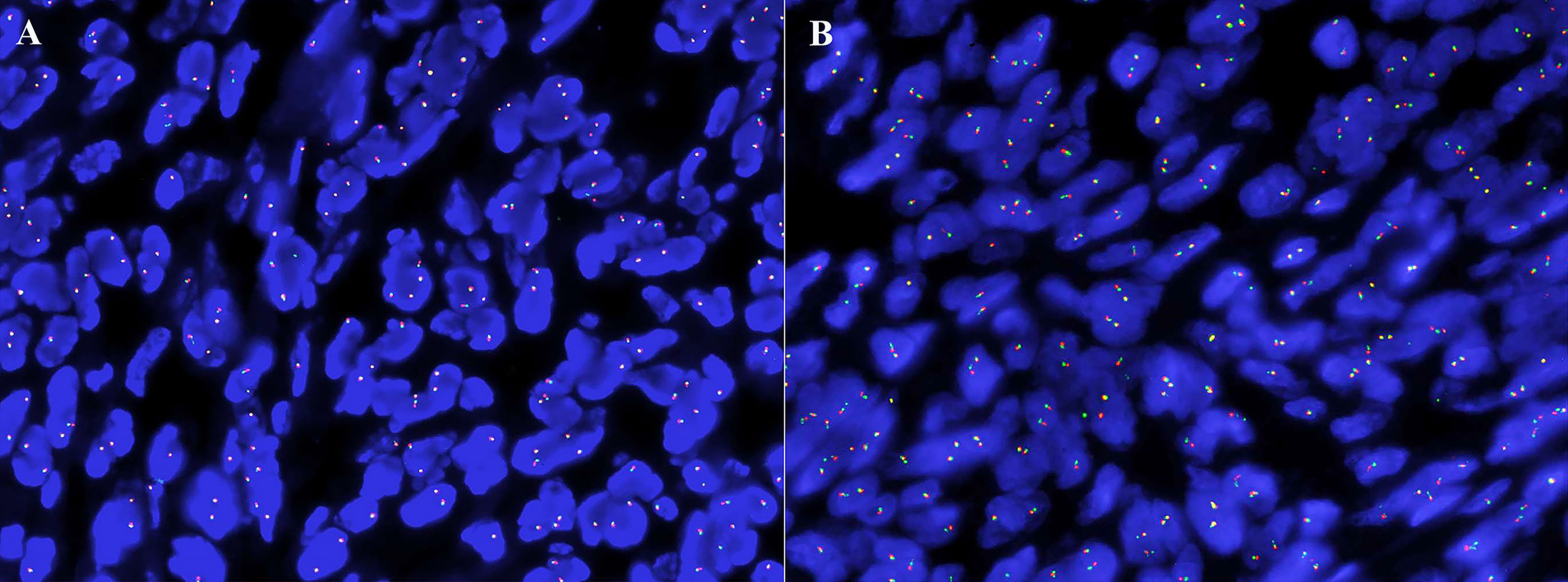
The absence of CSF1 **(A)** and USP6 **(B)** translocation in fluorescence *in situ* hybridization analysis.

The immunohistochemical analysis revealed that the synovium-like monocytes and xanthomatous cells were positive for Bcl-2 (**Figure 3D**), SMA (**Figure 3E**), CD68 (**Figure 3F**), and CD10. P63 was focally positive in mononuclear cells. Ki67 was positive in about 10%. The tumor cells were negative for desmin, CD34, S-100, EMA, PCK, TLE-1, CK5/6, HMB45, ALK, and CD30. In terms of fluorescence *in situ* hybridization (FISH) detection for the translocation of CSF1 gene and USP6 gene, the results were negative (**Figure 4**).

A diagnosis of GCTTS was made based on morphological and immunohistochemical characteristics and the results of FISH. As of this writing, the patient is still alive, without local recurrence or distant metastasis detected at 15 months into follow-up.

## Discussion

Primary giant cell tumors in the breast are not common. Nine studies had reported primary giant cell tumors of soft tissue (GCT-ST) arising in the breast (15–23). One case occurred in an elderly male, and the other 8 cases occurred in females. The age of the patients ranged from 36 to 74 years old (the median age was 59 years). The tumor sizes ranged from 2.5 to 13 cm (the median size was 3 cm). The physical examinations or image examinations showed well-circumscribed masses in 7 cases (17–21) and irregular-shaped masses in 2 cases (22, 23). Of the 8 cases with follow-up results, 1 patient died of lung metastasis after the initial presentation, and in the other 7 patients, local recurrence or distant metastasis did not occur (18). In all cases of GCT-ST arising in the breast, the use of FISH to detect CSF1 translocation was not performed. GCTTS is rarer compared with GCT-ST. In our case, a 39-year-old female presented with a well-circumscribed 3-cm mass, and the patient is still alive without local recurrence or distant metastasis after 15 months of follow-up. There was no difference in the growth pattern or clinical characteristics between breast primary GCT-ST and GCTTS.

The differential diagnoses of GCTTS include metaplastic carcinoma (MC), carcinoma with osteoclast-like stromal giant cells (OGCs), phyllodes tumor (PT), breast implant-associated anaplastic large cell lymphoma (BIA-ALCL) as well as soft tissue tumor such as GCT-ST, fibroma of tendon sheath (FTS), and synovial sarcoma (SS). The presence of ductal carcinoma *in situ* within the lesion and the evidence of epithelial differentiation could be observed by histopathological morphology and immunohistochemical results in both MC and carcinoma with OGCs. Although spindle stromal cells could be the main component in PT, the presence of leafy with epithelial-lined clefts and cellular fibroblasts could prompt the proper diagnosis. BIA-ALCL was characterized by a monotonous proliferation of malignant and large cells with positivity for CD30 and ALK. GCT-ST contains round/oval mononuclear cells and multinucleated osteoclast-like giant cells with similar nuclei. The stroma is vascularized. The tumor interstitium of GCT-ST contains calcification, interlaced metaplastic bone, and abundant blood vessels, which may lead to a blood lake with cystic hemorrhage. In some cases, mitoses ranged from 1 to 30/10 HPF. Additionally, the lesion in GCTTS shows more foamy-like histiocytes, Touton giant cells, and inflammatory cells mixed up with interstitial collagenization and accompanied by hemosiderin reaction or cholesterol crystallization than that of GCT-ST, and metaplastic bone is uncommon.

The most important and difficult thing is to differentiate GCTTS from cellular FTS and SS. Cellular FTS is composed of bland fibroblasts with elongated nuclei and dense eosinophilic collagen fibers. Slit-like vascular spaces are common in FTS. Compared with GCTTS, cellular FTS has less giant cells, xanthomatous cells, and hemosiderin. It is reported that USP6 translocation by FISH could serve as useful criteria for cellular FTS (24). Combining the histological characteristics and USP6 gene result, we excluded the diagnosis of cellular FTS. SS is a malignant soft tissue tumor most often found in the extremities of young adults. Spindle-shaped cells are arranged in herringbone and whorled patterns. Epithelial cells usually can be observed in spindle cell elements. Mitotic figures are visible. Most SS display immunoreactivity for cytokeratins, EMA, and TLE-1. The histological characteristics and immunohistochemical results helped us to exclude the diagnosis of SS. SS18 gene rearrangement by FISH could serve as useful criteria for SS, respectively, when the diagnosis is difficult (25).

Synovial metaplasia is a particular histological feature of this case. As previously reported, synovial metaplasia is one of the complications after breast augmentation (26). These metaplasia components have a similar morphology and transport capacity as with the synovial membrane (27). The mechanisms underlying the formation of synovial membranes might be a unique repair process (28). Some studies have described pigmented villonodular synovitis (PVNS) after knee arthroplasty (TKA), and the exact etiology of PVNS after TKA was considered relevant to polyethylene, trauma, and hemarthrosis (29, 30). In our case, GCTTS was diagnosed within 12 months after breast augmentation, which was similar to the case reported by Bunting et al. (29), supporting that operative procedure initiated the proliferative process. Combined with breast augmentation history, we concluded that the existence of synovial metaplasia of this patient was initiated by the proliferative repair process.

The pathogenesis of GCTTS in joint was thought to be related to the overexpression of CSF1, although it was first considered to be a reactive or hyperplastic process caused by inflammation (31). The translocation involved COL6A3 and CSF1, resulting in an overexpression of CSF1 (9). The secreted CSF1 fosters the accumulation of non-neoplastic mononuclear and multinucleated cells that form masses (8, 9). Detecting CSF1 translocation could assist in the diagnosis of GCTTS, and a total of 61–76% GCTTS displayed CSF1 translocations involving the 1p13 locus (8, 32). The CSF1 rearrangements in mononuclear cells create a landscaping effect in which most of the GCTTS contain non-neoplastic cells, which accumulate abnormally, and a smaller proportion of neoplastic cells (9). To explore the underlying molecular mechanism, CSF1 translocation was also assessed in this study. However, a significant CSF1 translocation was not detected in this rare case. Similarly, liposarcoma is the most common soft tissue sarcoma, but malignant PT and MC with heterologous liposarcoma are more common than primary liposarcoma in the breast (33). MDM2 amplification is a diagnostic criterion for liposarcoma in soft tissue (34), but at least 80% liposarcomatous differentiation in malignant PT and MC are unassociated with MDM2/CDK4 amplification (33). The studies showed that liposarcomatous differentiation within malignant PT and MC lacked the characteristic molecular alterations of soft tissue liposarcoma (35–37). In the same way, GCT-ST is a primary soft tissue neoplasm that is histologically similar to the GCT of bone (GCT-B) (38). It has been reported that >90% of GCT-Bs have a driver mutation in the H3F3A gene (39), but in a series of 15 GCT-ST, we found no H3F3A mutation in any case (38). According to the results, Lee et al. (38) believed that GCT-ST was probably genetically distinct from GCT-B. We hypothesized that, despite the histological similarity to GCTTS in arthrosis, the synovial metaplasia of capsules after implantation could cause GCTTS in the breast with the absence of CSF1 translocation. Despite the lack of CSF1 translocation, there may be other rearrangements leading to CSF1 upregulation in GCTTS. This CSF1/CSF1R signaling pathway needs to be further explored.

CSF1/CSF1R targeted therapy is an attractive therapeutic strategy for patients with symptomatic GCTTS associated with severe morbidity or functional limitations and not amenable to improvement with surgery (40). Pexidartinib is an orally available CSF1R inhibitor, which is the first systemic treatment approved by the US Food and Drug Administration in August 2019 (40). In the ENLIVEEN trial (NCT02371369), a randomized, double-blind phase III clinical trial showed that pexidartinib could improve the symptoms and function outcomes of patients with symptomatic GCTTS (41). The overall response of the pexidartinib group was higher than the placebo group at week 25 ENLIVEEN, and pexidartinib also significantly increased the relative range of motion and significantly improved the physical outcomes (41). ENLIVEEN showed that pexidartinib could induce serious and potentially fatal liver injury; mixed or cholestatic hepatotoxicity was an identified risk (41). A prospective observational study (NCT02948088) involving GCTTS patients receiving pexidartinib is now in progress (42). Moreover, several CSF1R inhibitors have been reported to act against GCTTS, including nilotinib, imatinib, and emactuzumab, and have shown encouraging results (43–45).

## Concluding Remarks

We presented a case of GCTTS in the breast after augmentation and concluded that the pathogenesis of this tumor based on synovial metaplasia was independent of classical CSF1 translocation. It was reported that a lower recurrence of GCTTS could be achieved by complete excision (46). Moreover, GCTTS should be included in the differential diagnosis of the lesion after breast augmentation.

### Limitation

The main limitation of this study is that only one patient was included.

## Data Availability Statement

The original contributions presented in the study are included in the article/supplementary material. Further inquiries can be directed to the corresponding author.

## Ethics Statement

Written informed consent was obtained from the individual for the publication of any potentially identifiable images or data included in this article.

## Author Contributions

ZZ and HB conceived and reviewed this case. YZ and YF collected the case details, wrote the article, and contributed equally to this manuscript. HZ provided and reviewed the cases. MC and JY were involved in providing technical support for molecular pathology. All authors contributed to the article and approved the submitted version.

## Funding

This research received funding from the Sichuan Science and Technology Program (no. 2022YFS0376). The funder subsidized the publishing expenses of this paper.

## Conflict of Interest

The authors declare that the research was conducted in the absence of any commercial or financial relationships that could be construed as a potential conflict of interest.

## Publisher’s Note

All claims expressed in this article are solely those of the authors and do not necessarily represent those of their affiliated organizations, or those of the publisher, the editors and the reviewers. Any product that may be evaluated in this article, or claim that may be made by its manufacturer, is not guaranteed or endorsed by the publisher.

## Abbreviations

GCTTS, giant cell tumor of tendon sheath; FISH, fluorescence *in situ* hybridization; GCT-ST, giant cell tumors of soft tissue; MC, metaplastic carcinoma; OGCs, osteoclast-like stromal giant cells; PT, phyllodes tumor; FTS, fibroma of tendon sheath; SS, synovial sarcoma; DCIS, ductal carcinoma *in situ*; PVNS, pigmented villonodular synovitis.
